# Multi-faceted exploration of the novel active γ-carbonic anhydrase PaCAγ1 in the human pathogen *Pseudomonas aeruginosa*

**DOI:** 10.1016/j.ijbiomac.2025.146755

**Published:** 2025-08-11

**Authors:** Vincenzo Massimiliano Vivenzio, Reygan Braga, Alessandro Bonardi, Vincenzo Alterio, Andrea Scaloni, Claudiu T. Supuran, Marianna A. Patrauchan, Giuseppina De Simone, Simona Maria Monti

**Affiliations:** a Istituto di Biostrutture e Bioimmagini, CNR, Via Pietro Castellino 111, 80131 Napoli, Italy; b Department of Environmental, Biological and Pharmaceutical Sciences and Technologies, University of Campania “Luigi Vanvitelli”, 81100 Caserta, Italy; c Department of Microbiology and Molecular Genetics, Oklahoma State University, Stillwater, OK 74078, United States; d Department of NEUROFARBA, Section of Pharmaceutical and Nutraceutical Sciences, University of Florence, Polo Scientifico, Via U. Schiff 6, 50019 Sesto Fiorentino, Firenze, Italy; e Proteomics, Metabolomics & Mass Spectrometry Laboratory, ISPAAM, National Research Council, 80055 Portici, Italy

**Keywords:** Antibiotic resistance, Antibacterial, Gamma-class carbonic anhydrase, Thermal stability and biophysical, characterization, Kinetic and inhibition, Macrophages, Biofilm

## Abstract

*Pseudomonas aeruginosa*, a ubiquitous Gram-negative bacterium, is an opportunistic pathogen that causes severe health issues in both immunocompetent and immunocompromised individuals. It is a member of the ESKAPE group of pathogens, and a leading cause of nosocomial infections worldwide. With the aim of identifying novel tools to combat *P. aeruginosa* infections, and considering that carbonic anhydrases are emerging as promising targets to overcome antibiotic resistance, we present here the identification and detailed characterization of a γ-class carbonic anhydrase, PaCAγ1, from *P. aeruginosa* using a multidisciplinary approach. Specifically, the identified enzyme was recombinantly produced and subjected to an extensive biochemical and biophysical characterization, proving its enzymatic function and revealing its overall stability at high temperatures. We determined that PaCAγ1 plays a role in pathogen fitness under carbon-limiting conditions and contributes to its survival within the macrophages. Various sulfonamide, sulfamide, and sulfamate derivatives were evaluated for their inhibitory properties against PaCAγ1 following a drug repurposing approach. This study reports on the discovery and characterization of a previously unrecognized γ-class CA, which enhances our understanding of the biochemical capacity and physiological importance of these enzymes in human pathogens.

## 1. Introduction

Carbonic anhydrases (CAs) are ubiquitous metalloenzymes that catalyze the reversible carbon dioxide hydration to bicarbonate and proton [1]. These enzymes are classified into eight genetically distinct families, α, β, γ, δ, ζ, η, θ, and ι [1–10], which preferentially contain a Zn (II) ion within the active site, but may also coordinate Co(II), Cd(II), or Fe(II) [8,11–13]. Only the latest discovered ι-class is metal-independent, performing the catalysis without any metal ion at its catalytic site and reflecting adaptation to metal limited environments [14]. Interestingly, although these enzymes perform the same catalytic function, their three-dimensional structure, as well as their oligomerization state differ from each other: α-class CAs are typically found as monomers and only occasionally as dimers [15–17], β-class CAs are dimers, tetramers or octamers [18–24], γ-CAs are trimers [25–27], ζ-CAs are normally monomers [8], θ-CAs are very similar to some β-CAs [10], while ι-CAs are dimers [2,14]. No information is available so far on the structural organization of δ- and η-CAs.

In humans, 15 isoforms of CAs are known, all belonging to the α-class [1]. They are expressed in a diverse range of tissues and regulate many important physiological processes, including the transport of CO_2_ from metabolizing tissues to the lungs for excretion [28], pH and CO_2_ homeostasis [29], and regulation of electrolyte secretion [30,31]. Unlike humans, bacteria encode CAs also belonging to β-, γ-, and ι-classes. The significant sequence and structural differences between these and α-class CAs, introduce an opportunity to specifically target bacterial CAs. For this reason, in the last decades, bacterial CAs have been investigated as possible targets for developing new antibacterial drugs [32–36], with a particular focus on the CAs identified in pathogenic bacteria with clinical significance. This strategy has been proven successful through targeting CAs of two antibiotic-resistant Gram-negative pathogens, *Neisseria gonorrhoeae* and *Helicobacter pylori*. For *N. gonorrhoeae*, treatments with CA inhibitors, acetazolamide (**AAZ**) and ethoxzolamide (**EZA**), showed long-lasting bacteriostatic effects with no apparent development of resistance [35,36]. Similarly, for *H. pylori*, **EZA** inhibited bacterial growth without triggering resistance [37]. Although more bacteria-specific inhibitors are needed, these results indicate that targeting CAs is a promising strategy for combating challenging bacterial pathogens.

*P. aeruginosa*, a ubiquitous Gram-negative bacterium, is an opportunistic pathogen that causes lethal infections in immunocompetent and immunocompromised individuals. Together with *Staphylococcus aureus*, *Escherichia coli*, *Streptococcus pneumoniae* and *Klebsiella pneumoniae*, it is ranked among the top five pathogens responsible for over half of bacterial infections-related deaths, globally [38]. *P. aeruginosa* infects different tissues, including lungs, wounds, ears, eyes, and urinary tract, where it produces a broad array of potent virulence factors that aid the pathogen invasion and survival within the host [39–44]. It can form biofilms, microbial communities enclosed within a self-produced protective matrix, that enhance resistance to both the immune system and antimicrobial treatments. This ability significantly improves fitness and resistance of the pathogen making the challenge of eradicating infections even more difficult [45]. Furthermore, *P. aeruginosa* possesses both intrinsic and acquired mechanisms of antibiotic resistance [45], leading to the frequent occurrence of resistant strains to practically all classes of antibiotics available today, such as β-lactams, quinolones, aminoglycosides, and colistin [46–50]. Given these challenges, there is a big effort in the scientific community to search for novel tools to combat *P. aeruginosa* infections, which includes the identification and validation of novel targets. Previously, three functional β-CAs were identified in *P. aeruginosa* PAO1 strain, namely psCA1, psCA2, and psCA3 [19,51–53]. Sequence analyses revealed that they are highly conserved among pseudomonads, and their occurrence in multiple homologs suggests a significant physiological role. The three enzymes are expressed under varying CO_2_ conditions, with psCA1, the most active, abundant, and particularly important for *P. aeruginosa* survival under low CO_2_ [51]. This enzyme showed a moderate activity with a k_cat_ of 1.8 × 10^5^ and a k_cat_/K_M_ of 7.5 × 10^7^ M^−1^ s^−1^ at pH 8.3 and was discovered to contribute to *P. aeruginosa* biofilm formation by enabling calcium deposition [52]. Among tested inhibitors, 3-amino-benzene suldonamide (**ABS**) and **AAZ** inhibited psCA1 catalytic activity with Ki of 19 and 37 nM, respectively. Further validating the role of the enzyme in calcium deposition, the latter was abolished upon treating *P. aeruginosa* with the CA inhibitors [52].

In this study we characterized a novel γ-class CA, named PaCAγ1, of the human pathogen *P. aeruginosa* PAO1 strain, providing the first extensive characterization of a γ-class CA in this bacterium. Results showed that PaCAγ1 has a pH-dependent catalytic activity and stability, and is effectively inhibited by nanomolar concentrations of various sulfonamide, sulfamide, and sulfamate derivatives. We also detailed its thermal stability and explored its potential physiological role in *P. aeruginosa*, particularly under different pH and carbon source conditions, as well as during phagocytosis.

## 2. Materials and methods

### 2.1. Identification, construct design, expression and purification of PaCAγ1

BLASTP sequence alignments using the National Center for Biotechnology Information (NCBI) non-redundant database (GenBank release 264.0) and known γ-CA amino acid sequences as a reference followed by Conserved Domain Database (CDC) prediction and conserved residues analyses predicted a putative γ-CA in the *P. aeruginosa* PAO1 genome encoded by PA5540, hereafter referred as PaCAγ1.

A synthetic gene encoding His-tagged PaCAγ1 was designed and commercially produced by GeneArt followed by its cloning into a pET21d plasmid. The resultant plasmid was transformed into *E. coli* BL21 (DE3) chemically competent cells. PaCAγ1 was recombinantly expressed in *E. coli* BL21 (DE3) grown in Luria-Bertani (LB) medium supplemented with 0.2 mg/mL ampicillin (Amp) at 37 °C. When the optical density at 595 nm (OD_595_) reached 0.6, isopropyl β-d-1-thiogalactopyranoside (IPTG) was added to a final concentration of 0.5 mM. After induction, the cells were allowed to grow for 2 h and harvested by centrifugation at 5000 ×*g* for 20 min, at 4 °C. The harvested cells were stored at −80 °C. Protein purification was carried out upon cell lyses in a buffer containing 20 mM TRIS, 100 mM NaCl, pH 8.5, supplemented with lysozyme, phenylmethylsulfonyl fluoride (PMSF), DNase, and 1× protease inhibitors (Sigma-Aldrich, Milan, Italy) and sonication at 4 °C. Following centrifugation at 14,000 ×*g* for 30 min at 4 °C, the total protein extract was loaded onto a 1 mL Cytiva His Trap HP column connected to ÄKTA pure system, which was equilibrated with a binding buffer consisting of 20 mM TRIS, 0.5 M NaCl, pH 8.5. The recombinant protein was eluted with 50 % v/v Elution Buffer (EB), consisting of 20 mM TRIS, 500 mM NaCl, 500 mM imidazole, pH 8.5, following a preliminary wash with 10 % v/v EB. The collected protein was subsequently concentrated using an Amicon Ultra-4 10 kDa cut-off filter and purified *via* size exclusion chromatography (SEC) using a Superdex 75 10/300 column 20 mM TRIS, 150 mM NaCl, pH 8.5 buffer.

### 2.2. Mass spectrometry measurements

Protein identity and purity was confirmed using a complete Surveyor HPLC system equipped with a C4 BioBasic column (ThermoElectron, Milan) connected on-line with a LCQ DecaXP Ion Trap mass spectrometer (ThermoElectron, USA) equipped with an OPTON electrospray source. PaCAγ1 was eluted with a 30 to 70 % gradient of solvent B (0.05 % trifluoroacetic acid in CH_3_CN) to solvent A (0.08 % trifluoroacetic acid in H_2_O) during 30 min. Mass spectra were recorded in a positive mode within the *m*/*z* range 400–2000 [54]. Deconvolution was carried out by means of the Magtran software 1.03 b2 [55].

### 2.3. Light scattering measurements

To evaluate the oligomeric state of PaCAγ1 in solution, SEC-MALS-QELS experiments were performed as previously described [56]. MALS-DAWN Treos^®^ and differential refractive index (dRI) Optilab^®^ instruments were connected online to the FPLC system. The experiment was conducted by loading 700 μg of purified protein onto the SEC s75 column using the above-mentioned SEC buffer, at a flow rate of 0.5 mL min^−1^; data were then analyzed by Astra software (Wyatt Technology).

### 2.4. Circular dichroism measurements

Secondary structure content of PaCAγ1 was evaluated by Circular dichroism (CD) analysis using a Jasco J-1500 spectropolarimeter equipped with a Peltier temperature control system. Sample 4.31 μM in 2 mM NaCl, 0.4 mM Tris pH 8.5 was used, and the corresponding spectrum was acquired in a range of 260–190 nm using a scan speed of 50 nm min^−1^ and a bandwidth of 1. CD signals were converted into mean molar ellipticity per residue (θ) in degrees cm^2^ dmol^−1^, and the collected data, obtained by a mean of quadruplicate, were analyzed by the DichroWeb website, setting CDSSTR as the reference method [57].

Thermal stability was analyzed in a temperature range of 20–100 °C with a temperature increase of 1 °C min^−1^, and the signal was monitored at 208 nm. Additionally, CD spectra were recorded every 10 °C in a range of 260–190 nm. CD spectra were also collected upon cooling back to 20 °C as well as after overnight storage at 4 °C.

A pH-dependent stability of PaCAγ1 was evaluated as follows: purified PaCAγ1 was concentrated at 8.4 mg/mL using an Amicon Ultra centrifugal filter unit with a 3 kDa molecular weight cut-off and then diluted to a final concentration of 0.15 mg/mL (7.18 μM) in buffers with varying pH: 5 mM sodium acetate (pH 4.0, 4.5, 5.0, 5.5), 15 mM sodium phosphate (pH 6.0, 6.5, 7.0, 7.4, 8.0), and 5 mM Tris-HCl (pH 8.5 and 9.0). Far-UV CD spectra were recorded using a Jasco J-1500 spectropolarimeter as previously described.

### 2.5. Dynamic light scattering measurements

To evaluate the hydrodynamic diameter of PaCAγ1, dynamic light scattering (DLS) measurements were carried out using a Malvern Zetasizer (Malvern, UK). The protein (90 μM) was filtered using a syringe-driven Whatman filter (0.1 μm cut-off), placed in a disposable cuvette and then analyzed at 25 °C. Data were calculated by a means of a trip-licate from the correlation function using Malvern technology software; only data with a Polydispersity value below 20 % were considered. For thermal stability study, 38 μM of protein was filtered as described above and analyzed at a temperature between 25 and 50 °C, increasing the temperature by 1 °C every minute, with an accuracy of 0.1 °C. A total of four data points was collected at each temperature.

### 2.6. Wilbur-Anderson assay and thermal stability measurements

To evaluate the CA activity of PaCAγ1, Wilbur-Anderson assay (WAU) was performed as previously described [58]. This method measures the time required for the catalyzed and uncatalyzed reactions to decrease the pH of the solution from 7.5 (blue) to 6.5 (light green) by monitoring absorbance at 570 nm. Bromothymol blue (pKi 7.1, Sigma, Aldrich, Milan, Italy) was used as pH indicator at a concentration of 95 μM. The experiment was performed using a Jasco V-730 spectrophotometer equipped with a Peltier temperature control system. Standards and blanks were included in each experiment. The protein activity was measured by the equation:

$$\text{WAU}=\left({\text{t}}_{0}-\text{t}\right)/\text{t}$$

where WAU is Wilbur-Anderson Unit, t_0_ and t are the time periods required for the blank and enzyme sample reaction, respectively, to reduce the absorbance corresponding to a pH shift from 7.5 to 6.5.

To determine the thermal stability of PaCAγ1 activity, thermal treatment was carried out in a BIO-RAD T100^™^ Thermocycler with a heating gradient of 0.1 °C/s, from 20 °C to target temperatures of 60, 80, and 100 °C. Following the treatment and cooling of the sample, residual CA activity was evaluated using the WAU method described above.

### 2.7. PaCAγ1 catalytic activity and inhibition measurements

Kinetic parameters of CO_2_ hydration activity of PaCAγ1 were acquired by using a stopped-flow instrument operating at the absorbance maximum of 557 nm, using the assay buffer 20 mM TRIS, pH 7.4, and 20 mM NaClO_4_ to maintain constant ionic strength [59]. Phenol red 0.2 mM served as an indicator. The initial rates of the CA-catalyzed CO_2_ hydration reaction were monitored for a period of 10–100 s. CO_2_ concentrations ranged from 1.7 to 17 mM. The kinetic parameters and inhibition constants were determined by using Lineweaver–Burk plots [60,61]. At least six traces of the initial 5 %–10 % of the reaction were used to determine the initial velocity. Uncatalyzed rates were determined similarly and subtracted from the total detected rates. Stock solutions of the inhibitor (10–100 mM) were prepared in distilled-deionized water, and dilutions up to 0.01 nM were subsequently made using the assay buffer. Inhibitor and enzyme solutions were mixed and preincubated for 15 min at room temperature to allow for the formation of the E-I (Enzyme-Inhibitor) complex or eventual active site-mediated hydrolysis of the inhibitor. Inhibition constants were obtained by nonlinear least-squares methods using equation for the non-competitive inhibition K_I_ = IC_50_ [62–65]. All the reported inhibition constants and kinetic parameters for the uninhibited enzyme are presented as the means calculated based on least three different determinations. All the tested sulfonamides were of the highest purity purchased from Sigma-Aldrich (Milan, Italy).

### 2.8. pH-dependent activity of PaCAγ1

PaCAγ1-catalyzed CO_2_ hydration reaction was monitored by stopped-flow kinetic method at different pH values (4.0 to 9.0) by measuring the *k*_*cat*_ parameter (n = 3). Buffer-indicator dye pairs included MOPS and bromocresol green (at pH 4.0–5.5) measured at a wavelength of 515 nm, MOPS and 4-nitrophenol (at pH 5.5–7.0) measured at a wavelength of 400 nm, HEPES and Phenol Red (at pH 7.0–8.0) measured at a wavelength of 557 nm, and Trizma^®^base and m-cresol purple (at pH 8.0–9.0) measured at a wavelength of 578 nm [66]. For each pH value, three technical replicates were performed. The results were expressed as mean ± standard deviation (SD).

### 2.9. Bacterial strains and media used for P. aeruginosa growth, and biofilm studies

The bacterial strains and plasmids used in this study are listed in Table 1. The cultures were stored in 10 % v/v skim milk at −80 °C. Wild type (WT) *P. aeruginosa* PAO1 strain was grown at 37 °C in the modified Biofilm Minimal Medium (BMM) [67] designated BMM8, which contained: 9.0 mM sodium glutamate, 50 mM glycerol, 0.08 mM MgSO_4_, 0.15 mM NaH_2_PO_4_, 0.34 mM K_2_HPO_4_, 145 mM NaCl, 200 μL trace metals, and 1 mL vitamin solution. Trace metal solution contained (per liter of 0.83 M HCl) 5.0 g ${\text{CuSO}}_{4}^{\cdot }{\text{5H}}_{\text{2}}\text{O}$, 5.0 g ${\text{ZnSO}}_{4}^{\cdot }{\text{7H}}_{\text{2}}\text{O}$, 5.0 g ${\text{FeSO}}_{4}^{\cdot }{\text{7H}}_{\text{2}}\text{O}$, and 2.0 g ${\text{MnCl}}_{2}^{\cdot }4{\text{H}}_{2}\text{O}$; vitamin solution contained (per liter): 0.5 g thiamine, and 1 mg biotin (GoldBio). The pH of the medium was adjusted to 7.0. When needed, the pH of the medium or the concentration of glycerol was modified. For DNA manipulations, *E. coli* and *P. aeruginosa* cultures were grown in Luria–Bertani (LB) broth (per liter: 10 g tryptone, 5 g yeast extract, 5 g NaCl) at 37 °C, with shaking at 200 rpm. Antibiotics used for *E. coli* were (per mL) 100 μg carbenicillin (Cb), 10 μg gentamycin (Gm), and for *P. aeruginosa*, 300 μg Cb, 60 μg Gm.

### 2.10. Generation of PaCAγ1 deletion mutant

**Fig. 1.**
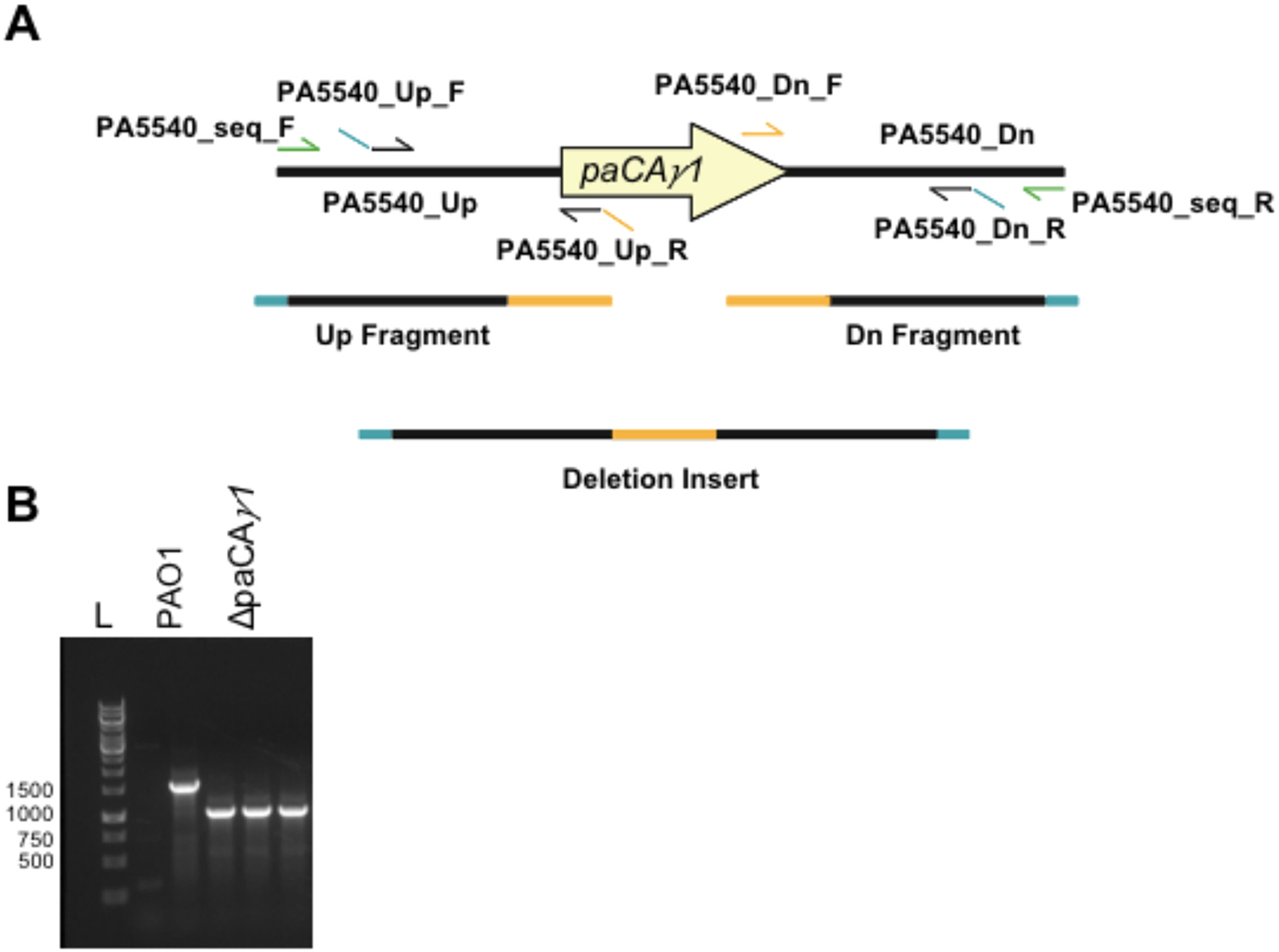
Deletion strategy and PCR verification of *paCAγ1* deletion. (A) Primer design for amplifying the upstream and downstream fragments of *paCAγ1*. Primers contain *attB* site sequences (blue) to facilitate allelic exchange. The amplified fragments were fused by overlap and fusion PCR techniques. (B) Deletion was verified by colony PCR using primers PA5540_seq_F/R and colonies of PAO1 and *ΔpaCAγ1*. The expected band sizes in PAO1 and *ΔpaCAγ1* are 1561 bp and 786 bp, respectively. Three colonies were tested. All primers with their sequences are listed in Table 1. L, ladder.

The deletion mutant *ΔpaCAγ1* was generated by using Gateway technology (two-step allelic exchange) as described in [68], with slight modifications. For this, three primer pairs were made as listed in Table 1. To facilitate allelic exchange, attB sequences were added to the 5′ end of each Up_F and Dn_R primer. The Up_R/Dn_F primers were designed to have an overlapping sequence at the beginning and the end of the gene targeted for deletion (Fig. 1A). All PCR reactions utilized Q5 polymerase with added Q5 GC enhancer (New England Biolabs). With the respective Up_F/Up_R and Dn_F/Dn_R primers, the upstream and downstream gene flanking segments were amplified. To assemble the amplified segments, an overlap extension PCR was ran followed by a fusion PCR, as detailed in [69]. The product was cloned into vector pDONRPEX18Gm using BP Clonase II (Invitrogen). The resultant plasmid, pRB003, was then transformed into E.coli DH5α cells using established heat shock protocol [69]; successful transformants were selected on LB agar plates containing Gm_10_ and verified by plasmid sequencing (Plasmidsaursus). After subsequent transformation into SM10 *E. coli* mating strain and PCR verification, biparental mating was conducted to introduce the construct into the PAO1 background as in [70]. Successful transformants were selected on *Pseudomonas* isolation agar (PIA) plates containing Gm_60_. To isolate a double crossover, colonies were grown on no-salt LB agar supplemented with 10 % *w*/*v* sucrose and selected using a SacB-based counter-selection. Sucrose^R^ and Gm^S^ mutants were selected and verified by PCR using seq_F/seq_R primers (Fig. 1B, Table 1).

### 2.11. P. aeruginosa growth studies

To elucidate the role of PaCAγ1 in *P. aeruginosa* physiology, growth studies were performed. *ΔpaCAγ1* and WT PAO1 were inoculated into 3 mL BMM8 and grown with shaking for 12 h at 37 °C. The cultures were normalized to an OD_600_ of 0.3 and inoculated into a 96 well-plate (1 % inoculum) with BMM8 containing either high (50 mM) or low (0.5 mM) glycerol at a pH of 5.4, 7.0, or 8.0. The plate was then placed into a Cytation5 plate reader, and OD_600_ was monitored every 2 h, for 24 h, with constant orbital shaking, at 37 °C.

### 2.12. P. aeruginosa survival in macrophages

To determine the role of PaCAγ1 in PAO1 survival in macrophages, J774.1 murine macrophages (ATCC) were cultured in Dulbecco’s Modified Eagle’s Medium (DMEM, Life technologies) supplemented with 10 % v/v heat-inactivated fetal bovine serum (FBS; Phoenix). Once cells were confluent (determined by phase-contrast microscopy), they were collected by manual scraping, counted utilizing a hemocytometer, normalized to 4 × 10^6^ cells/mL, then seeded into a 96-well plate (Corning) at a concentration of 2 × 10^5^ cells/well. The normalized precultures of PAO1 and *ΔpaCAγ1* (as in Section 2.11) were inoculated and grown for 12 h to middle-log phase (determined by OD_600_) in BMM8 supplemented with 5 mM glycerol, normalized to an OD_600_ of 0.2, and inoculated at a multiplicity of infection (MOI) of 10. Infected cells were incubated at 37 °C, 5 % CO_2_ for 30 min to allow uptake of *P. aeruginosa*. Following the incubation, the infected macrophages were pelleted at 2201 g for 2 min, after which 100 μL DMEM supplemented with 200 μg/mL Gm was added to each well to kill extracellular bacteria. After 30 min of treatment, samples (representing the initial bacterial load) were lysed with water and serially diluted for colony forming unit (CFU) quantification. To determine *P. aeruginosa* survival, the parallel samples were incubated in the presence of 200 μg/mL Gm for a total of 3 or 5 h. After incubation, macrophages were lysed, and the lysates were serially diluted for CFU. The percent of *P. aeruginosa* survival was calculated by using the initial and the final CFU/ml for 3 or 5 h post infection (hpi) for each respective strain. Graphs depict the average of three biological replicates, each including two technical replicates. Each experiment was repeated at least twice for consistency.

### 2.13. P. aeruginosa biofilm formation

Biofilm formation was assessed in both PAO1 and *ΔpaCAγ1* by fluorescent microscopy and crystal violet (CV) staining [71]. For this, cultures were grown shaking at 37 °C in 3 mL BMM8 supplemented with 0.5 mM glycerol at pH 7 or 5.4 for 12 h. Following normalization to an OD_600_ of 0.3, they were inoculated (1 %) into 6-well plates for microscopy or 96-well plates for CV assays. For microscopy, prior to inoculation, glass coverslips (Platinum Line^R^) were placed into each well. The plates were then incubated statically at 37 °C for 18–24 h, after which the coverslips were washed with saline to remove loosely attached cells. The biofilms were stained with 60 μL of Hoechst DNA stain solution, consisting of 56 μL saline and 4 μL of Hoechst (Invitrogen), for 1 h at room temperature in the dark, washed again and mounted onto slides for observation using Leica DMi8 fluorescent microscope [52]. For the CV assay, 180 μL of the inoculated (1 %) cultures were added to 96-well plate along with respective BMM8 negative controls and incubated for 24 h statically at 37 °C. Subsequently, 150 μL of the planktonic cultures were transferred to new wells to measure OD_600_ as a growth control. Then the wells were gently washed with saline to remove loosely associated cells and stained with 200 μL of 0.1 % w/v CV in 33 % acetic acid for 15 min. Upon thorough washing, CV was extracted with 200 μl of 95 % ethanol, and absorbance at 590 nm was measured. Each experiment included four biological replicates and was repeated at least three times for consistency.

## 3. Results

### 3.1. Identification of PaCAγ1 and genomic neighborhood of its encoding gene

**Fig. 2.**
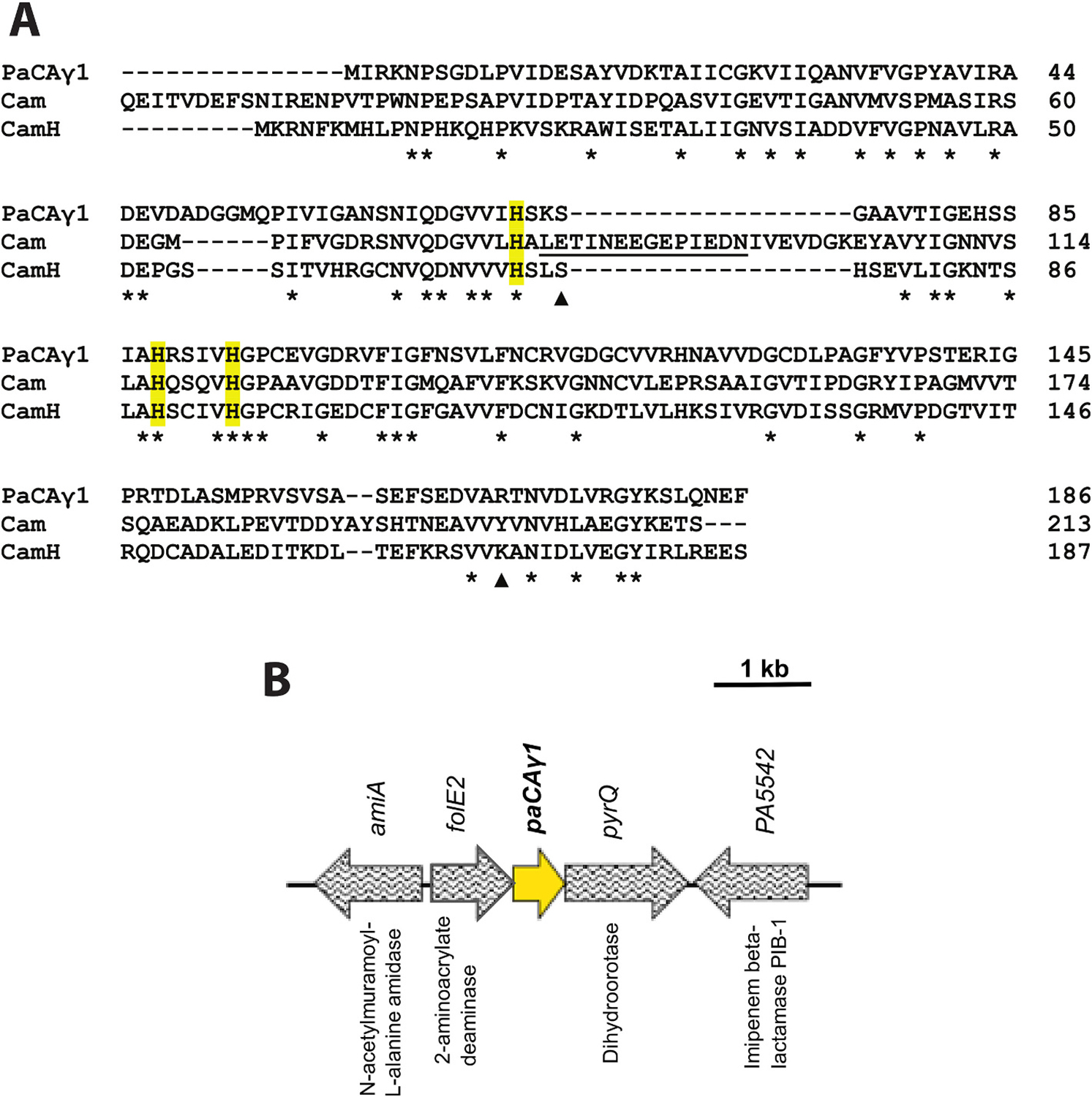
Multiple sequence alignment of PaCAγ1 with Cam and CamH γ-CAs and genomic neighborhood. (A) PaCAγ1 amino acid sequence was aligned with Cam (PDB: 1THJ) and CamH (UniProtKB ID: C3W4Q7) from *Methanosarcina thermophila*. Highlighted in yellow are the three catalytic histidines, whereas asterisk indicates strictly conserved residues. Black triangles highlight the key Cam residues E84 and Y200 not conserved in PaCAγ1. The acidic loop of Cam is underlined. Alignment was performed using Clustal W. (B) Genomic neighborhood of *paCAγ1* (*PA5540*), gene names and functions are as in PAO1 genome assembly (pseudomonas.com). The *paCAγ1* is represented by the yellow arrow. Arrows depicting a chevron pattern represent enzymes.

In this work, we aimed to identify and characterize a novel γ-CA in the human pathogen *P. aeruginosa* potentially exploitable as a target for novel antibacterial treatments. BLASTP sequence alignments using the two known γ-CAs, YrdA from *E. coli* [27] and Cam from *Methanosarcina thermophila* [25], identified a putative γ-CA sequence with the gene identifier PA5540 in the *P. aeruginosa* PAO1 genome. Hereafter the protein is referred to as PaCAγ1. The multiple sequence alignment of PaCAγ1 with the first structurally characterized γ-CAs, namely Cam and CamH from *Methanosarcina thermophila* (Fig. 2A), was consistent with the presence of the three catalytic histidines fundamental for the enzymatic function.

To gain insight into the role of PaCAγ1, the genomic neighborhood of its encoding gene was analyzed (Fig. 2B). In contrast to the previously identified γ-CA PaaY in *Acinetobacter baumannii*, whose gene lies within the *paa* gene cluster and plays a role in the phenylacetic acid (PAA) catabolic pathway as a thioesterase [26,72,73], the gene encoding PaCAγ1 (PA5540) is situated within a predicted operon harboring a predicted GTP cyclohydrolase (PA5539 — *folE2*) and dihydroorotase (PA5541 — *pyrQ*). The GTP cyclohydrolase FolE2 catalyzes the initial step of the *de novo* tetrahydrofolate biosynthetic pathway, essential for the production of nucleotides and amino acids [74,75], while *pyrQ* encodes a dihydroorotase responsible for the conversion of carbamoyl aspartate into dihydroorotate, a key step in pyrimidine biosynthesis pathway [76,77]. Since bicarbonate is required for carbamoyl aspartate synthesis, we hypothesized that PaCAγ1 supports pyrimidine biosynthesis by catalizing the conversion of CO_2_ into bicarbonate in the molecular proximity of this pathway. Framing the operon, there are genes encoding a *N*-acetylmuramoyl-L-alanine amidase, involved in peptidoglycan catabolism during cell division (*amiA*) [78], and the beta-lactamase PIB-1 [79] (Fig. 2B). This more distant genomic-neighborhood may expand the functional role of PaCAγ1 to include cell wall biosynthesis and resistance to beta-lactams. It is worth noting that the gene cluster from PA5532 to PA5541 has been reported to be regulated by Zur, a global regulator of Zn^2+^ acquisition under Zn^2+^ depletion [76] which may reflect the Zn^2+^ dependence of PaCAγ1 as shown for other CAs [80].

### 3.2. Design, expression, purification and biophysical characterization of PaCAγ1

**Fig. 3.**
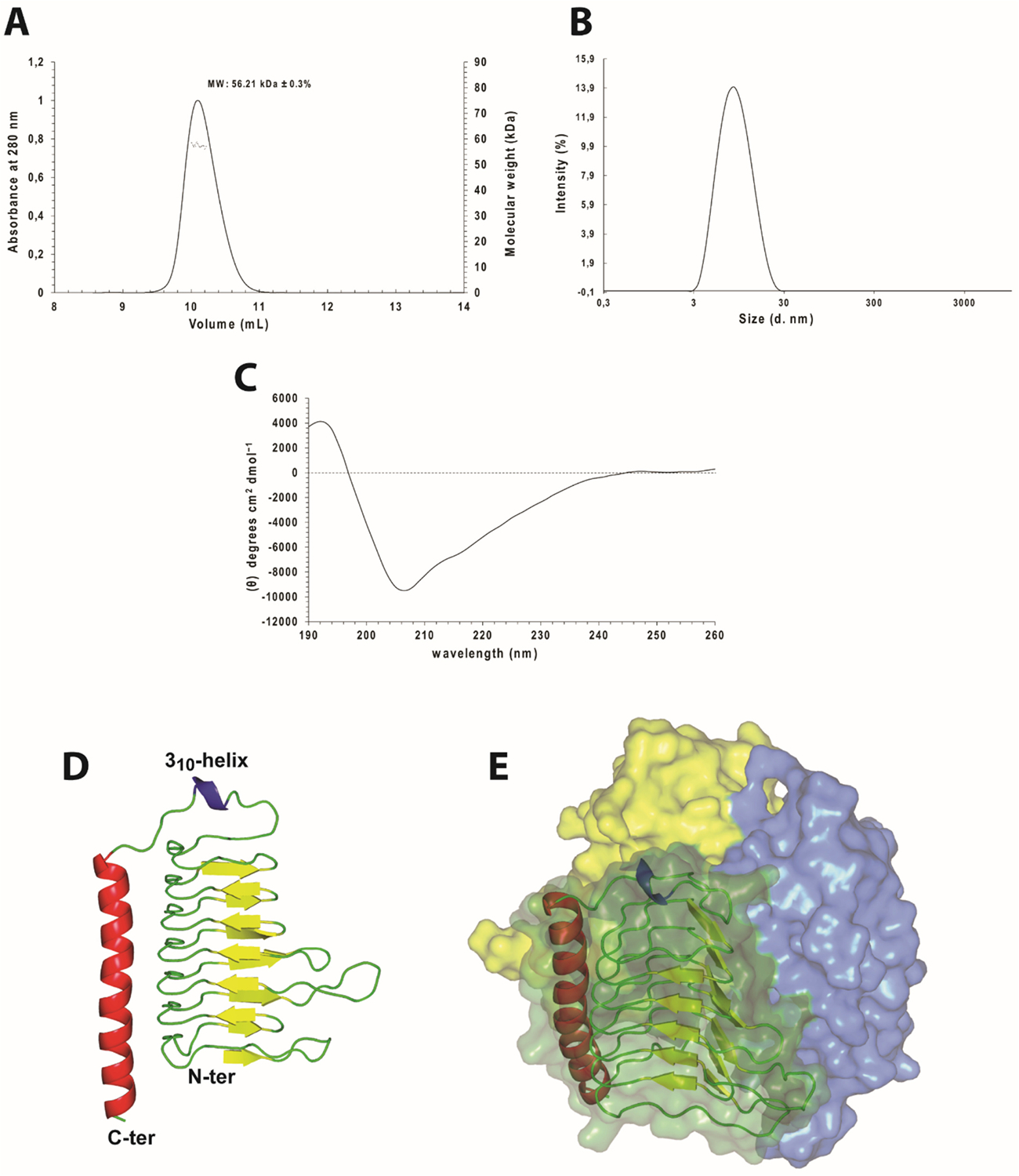
Biochemical and biophysical features of PaCAγ1. (A) PaCAγ1 forms a trimer according to SEC-MALS-QELS. (B) DLS measurements acquired at 25 °C. (C) Native CD spectrum recorded at 20 °C in 10 mM phosphate buffer, pH 7.4 at 4.3 μM concentration. (D) AlphaFold2 3D model of the PaCAγ1 monomer. Secondary structure elements are highlighted with the following color scheme: α-helix in red, β-sheet in yellow, loops in green and 3_10_-helix in blue. N- and C-terminal ends are explicitly indicated. (E) Surface representation of PaCAγ1 trimer generated with ColabFold v1.5.5, showing the three monomers in green, yellow and blue, respectively. One monomer is represented as a semitransparent surface to show the cartoon representation.

A his-tagged *paCAγ1* synthetic gene was designed and cloned into the commercially available pET21d plasmid, which allowed the expression of PaCAγ1 with a 6× His-tag at the C-terminus. Following the expression and purification protocols described in the Materials and methods section, PaCAγ1 was purified at a purity level greater than 98 % (according to SDS-PAGE) with a final yield of 5 mg/L. LC-MS analysis confirmed the protein identity and purity showing one main peak at 20,762.60 Da corresponding to the full-length PaCAγ1 protein including the C-terminal His-tag, but lacking the starting N-terminal methionine (Fig. S1) (MW_theoretical_ = 20,893.56 Da). PaCAγ1 was eluted by SEC as a unique peak with a retention volume of 10.1 mL, which is indicative of an oligomeric assembly. This data was confirmed by SEC-MALS-QELS which showed that the protein was present in solution as a homogenous trimer with a molecular mass of 56 kDa ± 0.3 (Fig. 3A) and a hydrodynamic diameter (D_H_) of 8.07 ± 0.19 nm (Fig. 3B). The secondary structure content of PaCAγ1 was evaluated by far UV-CD experiments. The collected spectra at 20 °C indicated a native conformation of the enzyme characterized by a pronounced negative peak at 208 nm and a positive peak at 192 nm (Fig. 3C). The secondary structure content estimated by DichroWeb was 14 % α-helix and 31 % β-sheet. These results align with the secondary structure content predicted for PaCAγ1 by the AlphaFold2 model. Additionally, the 222/208 nm ratio of 0.5, along with the reduced intensity of the band near 195 nm compared to the typical signal of α-helices, suggested the presence of 3_10_ helices (Fig. 3D) [81] consistent with the prediction of the AlphaFold2 model [82].

### 3.3. Effect of temperature on PaCAγ1 stability

**Fig. 4.**
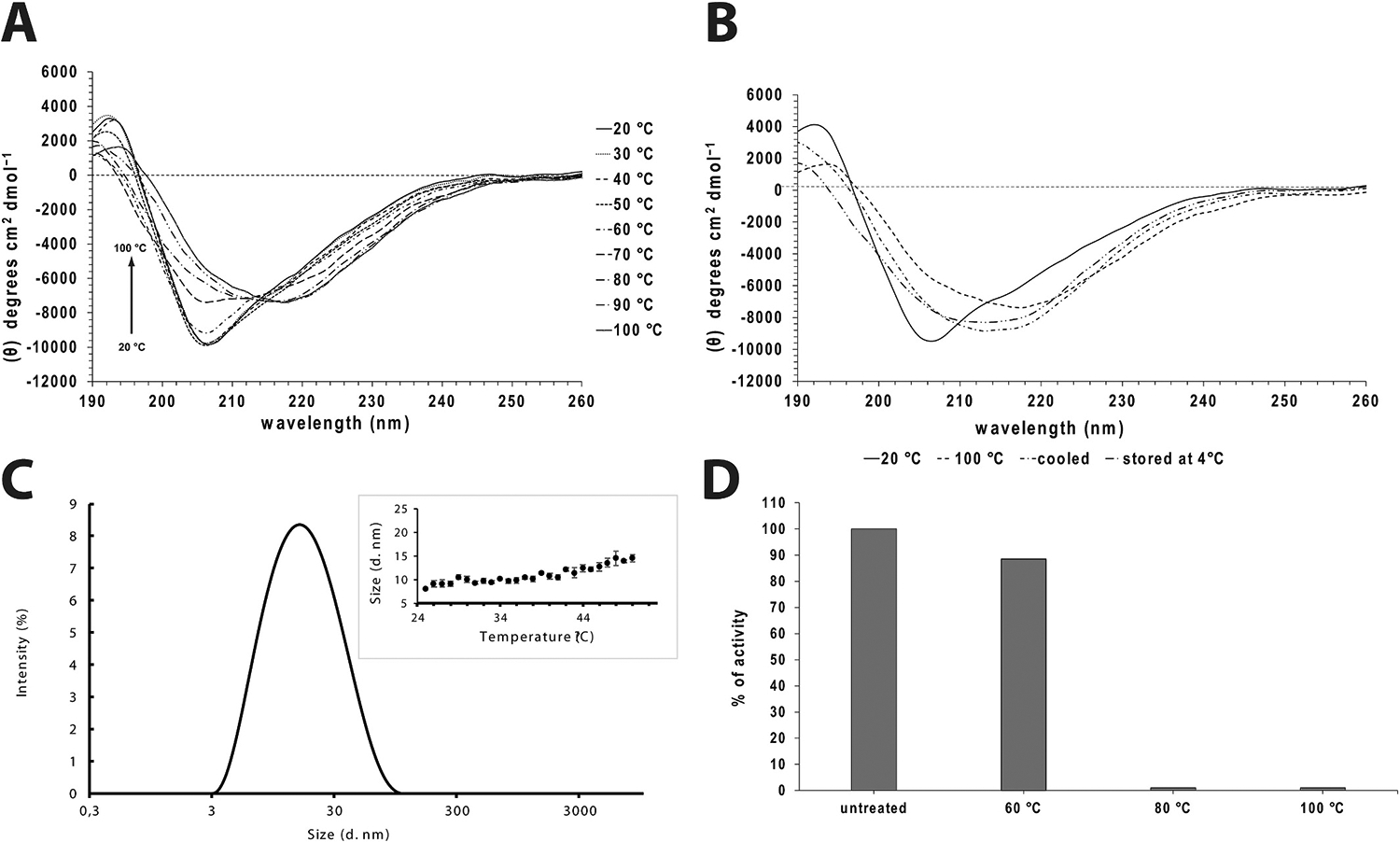
PaCAγ1 thermal stability. (A) Temperature effect on PaCAγ1 folding at 20 °C, 30 °C, 40 °C, 50 °C, 60 °C, 70 °C, 80 °C, 90 °C and 100 °C. (B) Overlay of far-UV CD spectra at 20 °C (solid line), 100 °C (dashed line), and cooled to 20 °C again (dash-dot line), and upon overnight storage at 4 °C (dash-dot-dot line). (C) D_H_ of PaCAγ1 after heating at 50 °C. Insert: D_H_ calculated at each temperature from 25 to 50 °C. (D) Assessment of PaCAγ1 activity upon thermal treatment at 60, 80 and 100 °C presented as % of the activity compared to the untreated protein.

As part of a comprehensive biochemical characterization of the enzyme and considering that other γ-CAs exhibited considerable thermal stability [26,66], we investigated the potential impact of temperature on PaCAγ1 stability by CD, monitoring changes in ellipticity upon heating the sample (Fig. 4A). No conformational changes were observed up to 60 °C, whereas a slight modification was observed at 70 °C, with a reduction of the intensity of the signal at 208 nm being the most apparent change. To investigate whether a loss of secondary structure could be observed at higher temperature, spectra were also collected at 80 °C, 90 °C, and 100 °C. In the latter conditions, a slight conformational rearrangement consisting of a further small reduction of the signal at 208 nm and a slight increase in the signal at 222 nm were observed (Fig. S2). Interestingly, even at these very high temperatures, the CD spectra did not resemble those typical of a denatured protein. These conformational alterations were maintained after cooling the sample and returning to the initial temperature of 20 °C (Fig. 4B). These data indicate that PaCAγ1 is a thermostable macromolecule that retains its secondary structure contents at temperatures up to 60 °C. At higher temperatures (80 °C and 100 °C), localized denaturation occurs in certain structured regions of the protein, suggesting partial unfolding. Thermal stability was also investigated by DLS measuring variations in hydrodynamic diameter upon temperature increase from 25 °C to 50 °C. In this range, no temperature-induced oligomeric dissociation was revealed with a D_H_ increase to 13.94 ± 0.29 nm during thermal treatment (Fig. 4C).

### 3.4. CO_2_ hydration assay and temperature effects on CA activity

The initial characterization of the catalytic activity of PaCAγ1 was carried out by the WAU assay [58] tracking the time required for the pH value to drop from 7.5 to 6.5, based on the reduction in absorbance at 570 nm of bromothymol blue. In the tested experimental conditions, PaCAγ1 displayed CO_2_ hydration activity of 25 WAU/mg, proving the catalytic activity of the protein.

The WAU assay was also used for monitoring the effect of temperature on the catalytic activity of the enzyme. In particular, PaCAγ1 was subjected to thermal treatment using a gradient of 0.1 °C/s, heated from 20 °C to 60 °C, 80 °C and 100 °C. Interestingly, the sample heated up to 60 °C retained 89 % activity, and only heating to 80 °C and 100 °C caused a complete loss of the activity compared to the untreated sample (Fig. 4D). In accordance with other γ-CAs previously studied [66,83,84], PaCAγ1 did not exhibit any esterase activity (data not shown).

### 3.5. Kinetic parameters and pH-dependence stability and activity of PaCAγ1

**Fig. 5.**
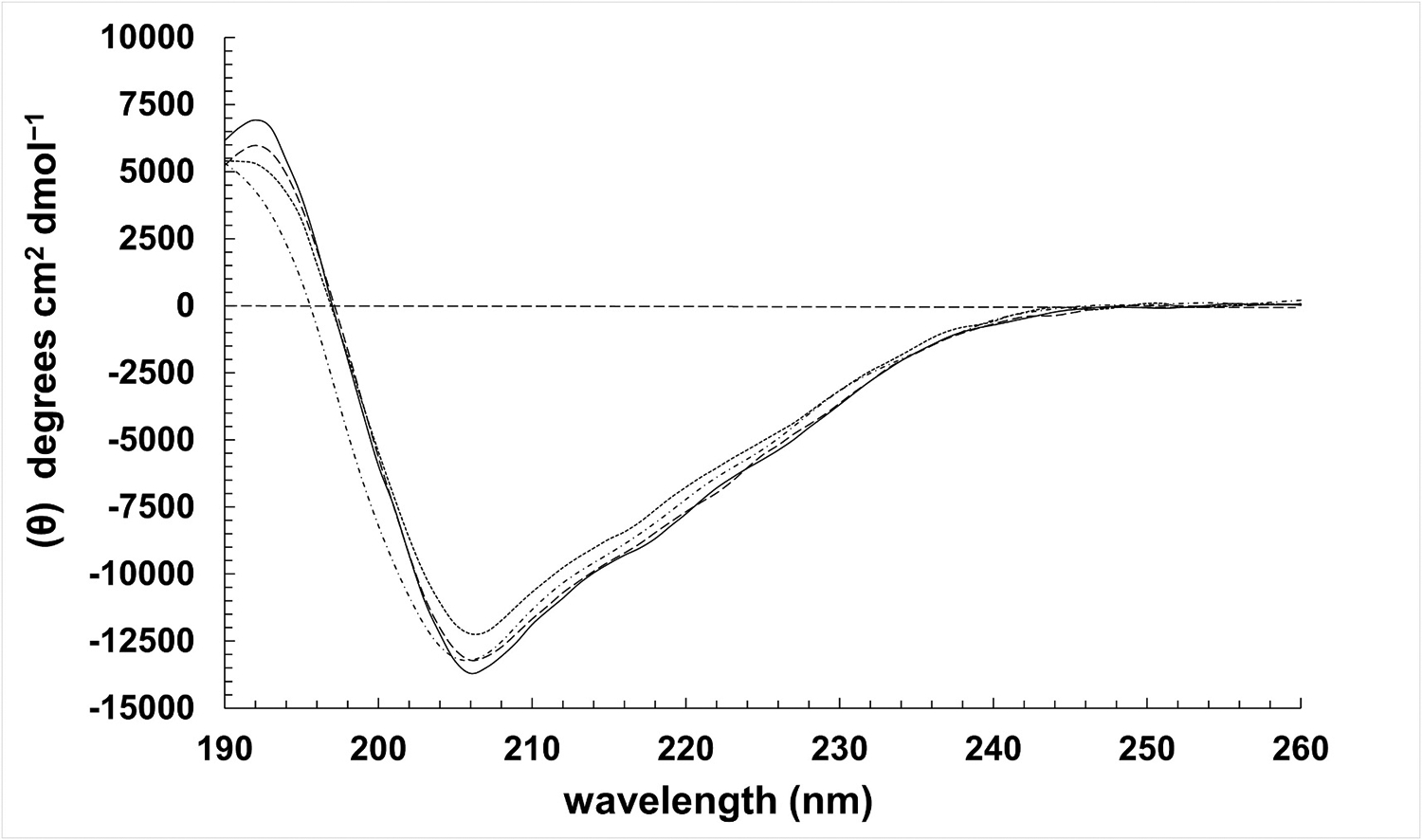
pH stability of PaCAγ1. Overlay of far-UV CD spectra at 20 °C of PaCAγ1 at different pH values: pH 7.0 (dashed line), pH 7.4 (solid line); pH 8.0 (dotted line), and pH 8.5 (dash-dot line).

A detailed kinetic characterization of PaCAγ1 enzymatic activity was carried out by stopped flow method at pH 7.4 showing that PaCAγ1 has a catalytic efficiency (k_cat_/K_M_) of 4.05 ×10^7^ M^−1^ s^−1^, a turnover number (k_cat_) of 4.70 ×10^5^ s^−1^, and an affinity for the substrate (K_M_) of 11.6 mM (Table 2). These values fall within the range reported for other γ-CAs, such as EcoCAγ (γ-CA from *E. coli*, k_cat_ of 5.7 ×10^5^ s^−1^ and k_ca_t/K_M_ of 6.9 ×10^6^ M^−1^ s^−1^) [85], BpsCAγ (γ-CA from *B. pseudomallei*, k_cat_ of 5.3 ×10^5^ s^−1^ and k_cat_/K_M_ 2.5 ×10^7^ M^−1^ s^−1^) [86] and VchCAγ (γ-CA from *Vibrio cholerae*, k_cat_ of 7.4 ×10^5^ s^−1^ and k_cat_/K_M_ 6.4 ×10^7^ M^−1^ s^−1^) [87]. The stability of PaCAγ1 as a function of pH was investigated by CD across a broad pH range (4.0–9.0). The enzyme retains its secondary structure content between pH 7.0 and 8.5, as indicated by minimal variations in the CD spectra (Fig. 5), suggesting that PaCAγ1 remains structurally stable and properly folded within this range. At pH values below 7.0 or above 8.5, the enzyme exhibits a loss of ordered secondary structure, as evidenced by a shift in the zero-crossing point of the spectra and a general reduction in signal intensity, indicating a reduced stability (Fig. S3). Accordingly, the catalytic activity measured at different pH values shows an inactive enzyme at pH below 6.5 and a reduced activity at pH higher than 8.0 (Fig. S3).

### 3.6. Inhibition profile of of PaCAγ1

**Fig. 6.**
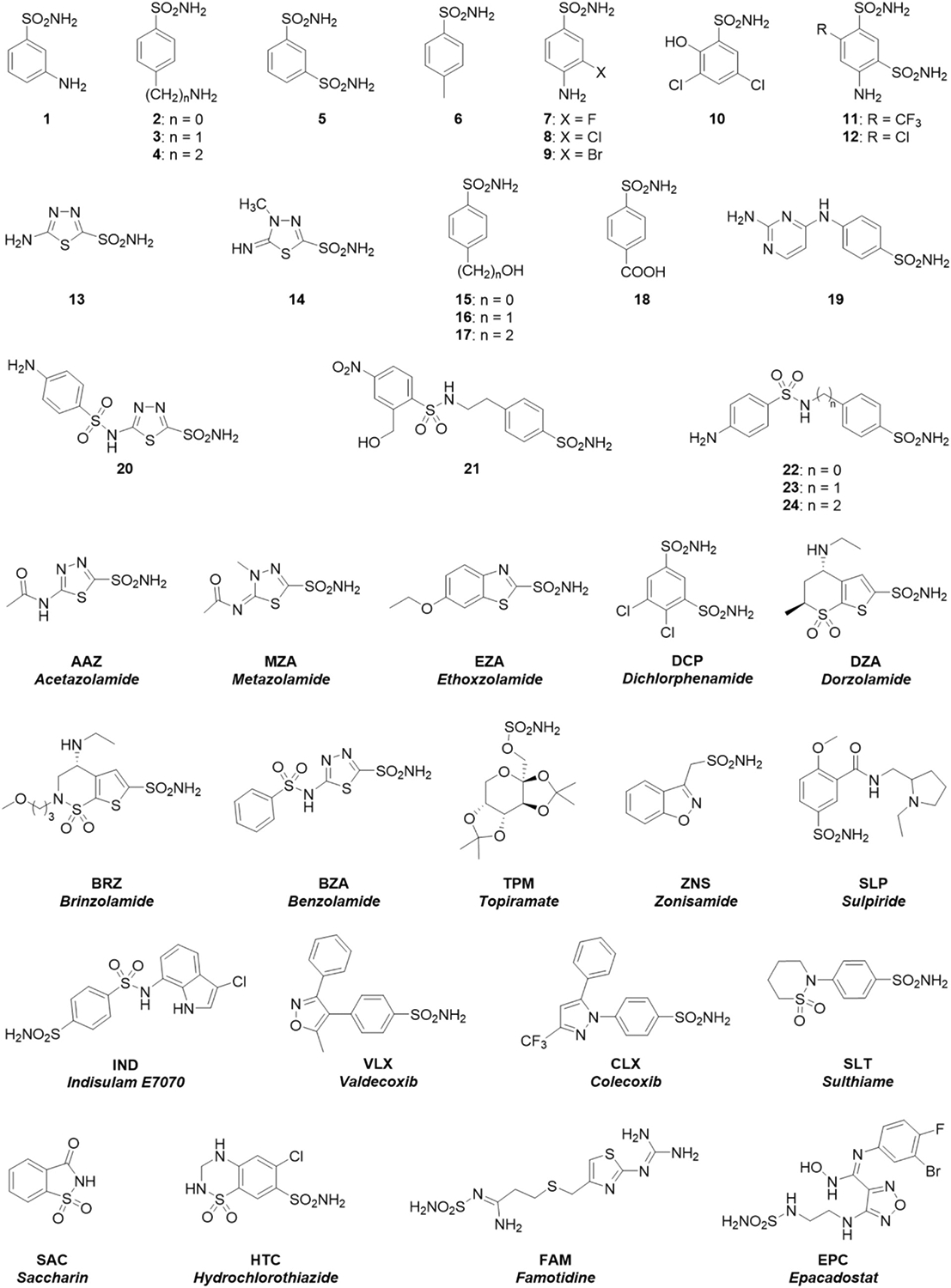
Inhibitors. The structures of sulfonamides, sulfamides, and sulfamates (**1–24** and **AAZ-EPC)** tested as inhibitors of PaCAγ1 in the present study.

The inhibition profile of the enzyme with a set of compounds **1–24** and **AAZ-EPC** sulfonamides, sulfamides, and sulfamates (Fig. 6) was also investigated. The inhibition constants (*K*_I_) for PaCAγ1 are presented in Table 3 alongside those for the human isoforms hCA I and II, as well as the corresponding Selectivity Index (*SI*) values for comparison. Based on the structural features of the inhibitors and their activity against PaCAγ1, we derived the following aspects of structure-activity relationship (SAR):

- The most potent PaCAγ1 inhibitors included clinically used **AAZ** and **MZA**, along with their deacetylated precursors **13** and **14**, showing *K*_I_ values in the low nanomolar range of 47.2–97.1 nM. Notably, deacetylation decreased potency by about 1.5 times, suggesting the importance of the acetyl group in interacting with PaCAγ1 active site residues. Even though the precursors are less effective against hCA I and hCA II, compounds **13** and **14** are 118 and 95-fold, respectively, more potent against PaCAγ1 than against hCA I.
- Several derivatives, such as **1–8**, **15–18**, **20–24**, **EZA**, **DZA**, **BRZ**, **BZA**, **IND**, and **SLT**, have lower potency, albeit with *K*_I_ values <1000 nM. Compounds **1**, **2**, and **4** were the most selective for PaCAγ1, with selectivity ratios ranging from 148 to 336 times compared to hCA I and 1.4–1.6-fold compared to hCA II. In contrast, compounds **5–8**, **15–17**, **DZA**, and **BRZ** exhibited affinity against the hCA isoforms in the order hCA II > PaCAγ1 > hCA I, with a hCA I/**PaCAγ1** selectivity ratio ranging from 14 to 295-fold. It is challenging to rationalize the SAR in this case, as these compounds belong to highly diverse classes of sulfonamides with both aromatic and heterocyclic derivatives. However, it appears that *p*-substituted aromatic sulfonamides serve as better inhibitors than *m*-substituted compounds (*e.g*., in **2–4** compared to **1** and **5**), while the incorporation of bulkier substituents (*e.g*., in **5** and **8** compared to **1** and **2**, or **BRZ** compared to **DZA**) impairs inhibitory activity. Furthermore, heteroaromatic sulfonamides tend to exhibit a greater potency than benzene-sulfonamides, as in **13**, **12**, and **20** compared to **2** and **22**, or in **EZA**, **DZA**, **BRZ**, and **BZA** compared to **IND**, and **SLT**.
- Compounds exhibiting low micromolar *K*_I_ values for PaCAγ1 included **9–12**, **19**, **DCP**, **ZNS**, **HTC**, **FAM**, and **EPC**, with *K*_I_ values ranging from 1.3 to 9.6 μM. Similar to the above discussed subset, these compounds belong to many diverse chemotypes, making it difficult to rationalize their SAR. Notably, derivatives **9**, **11**, **12**, and **EPC** were 1.4 to 1.9-fold more potent against PaCAγ1 than hCA I.
- **SAC** inhibited PaCAγ1 with a *K*_I_ of 13.8 μM, which makes it 1.3-fold more efficient compared to hCAI.
- Overall, the inhibition profile of PaCAγ1 and hCA I/II diverged significantly due to differences reflecting their belonging to distinct CA families. This distinction is also underscored by the inhibition profile of **TPM**, **SLP**, **VLX**, and **CLX**, with present *K*_I_ values exceeding 100 μM against the bacterial enzyme. Their bulky molecular structure may fit better into the conical α-CA active site than the smaller γ-CA active site.

### 3.7. P. aeruginosa growth studies

**Fig. 7.**
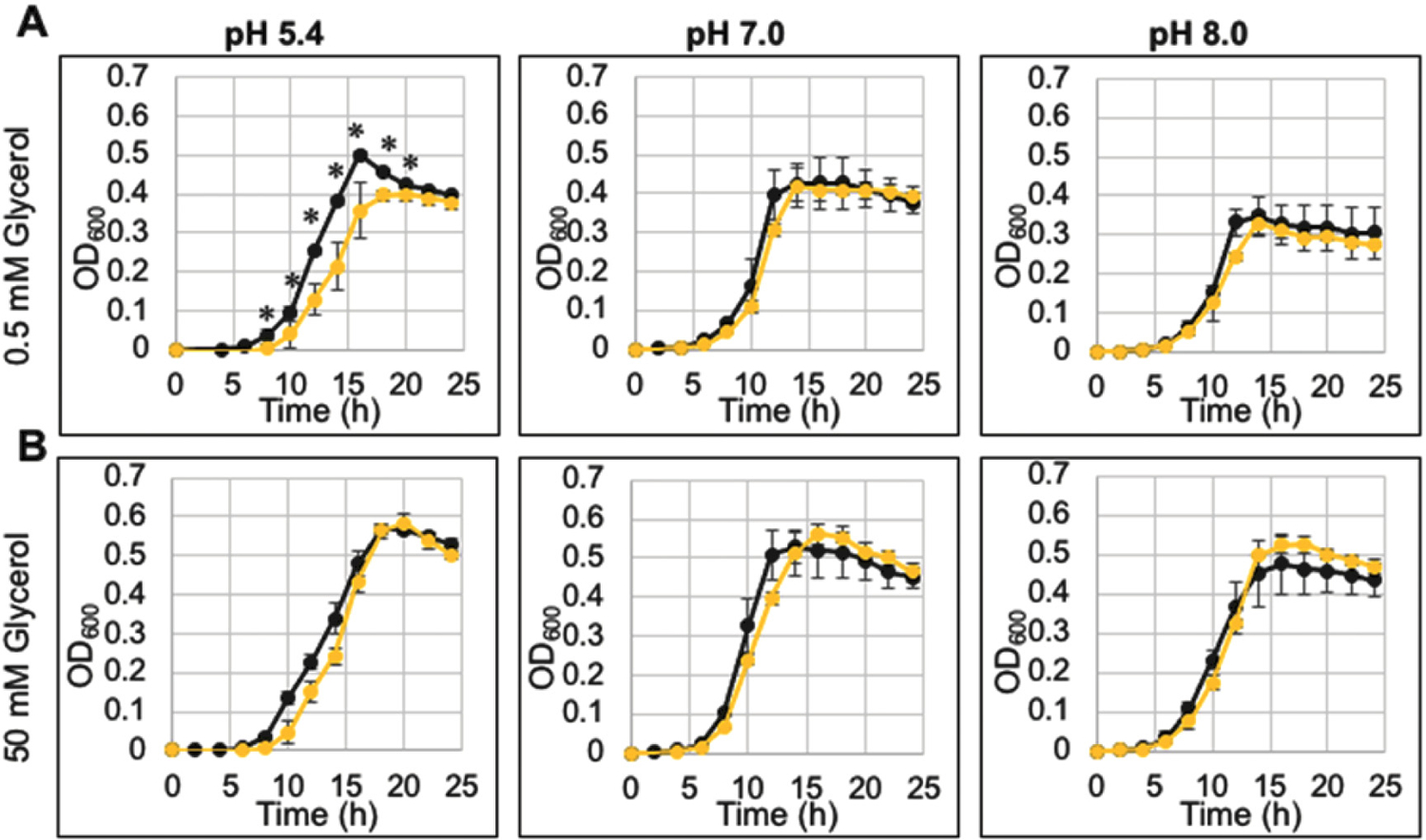
Growth of *P. aeruginosa* PAO1 and *ΔpaCAγ1* strains at 37 °C under shaking conditions. The *ΔpaCAγ1* mutant (yellow) was grown alongside WT PAO1 (black) in BMM8 medium containing either 0.5 mM glycerol (A) or 50 mM glycerol (B) at pH 5.4, 7.0, and 8.0. Statistical significance was determined using a single factor ANOVA * indicates *p* < 0.05.

Given that the genomic context indicated a potential involvement of PaCAγ1 in central anabolic pathways, we hypothesized that the enzyme may contribute to *P. aeruginosa* fitness. To test this hypothesis, we monitored growth of *ΔpaCAγ1* mutant alongside WT at various conditions. When grown shaking at 37 °C, a growth defect of *ΔpaCAγ1* was observed in low carbon (0.5 mM glycerol) medium at a pH of 5.4 (Fig. 7A). This pH value was chosen to reflect the increased acidity during phagosome maturation [88,89]. However, at higher carbon levels (50 mM glycerol) at pH 5.4 or at pH 7.0 and 8.0, the latter two representing pH during bacterial colonization of skin and chronic wounds [90], no difference in growth was observed (Fig. 7A,B).

### 3.8. P. aeruginosa survival in macrophages

**Fig. 8.**
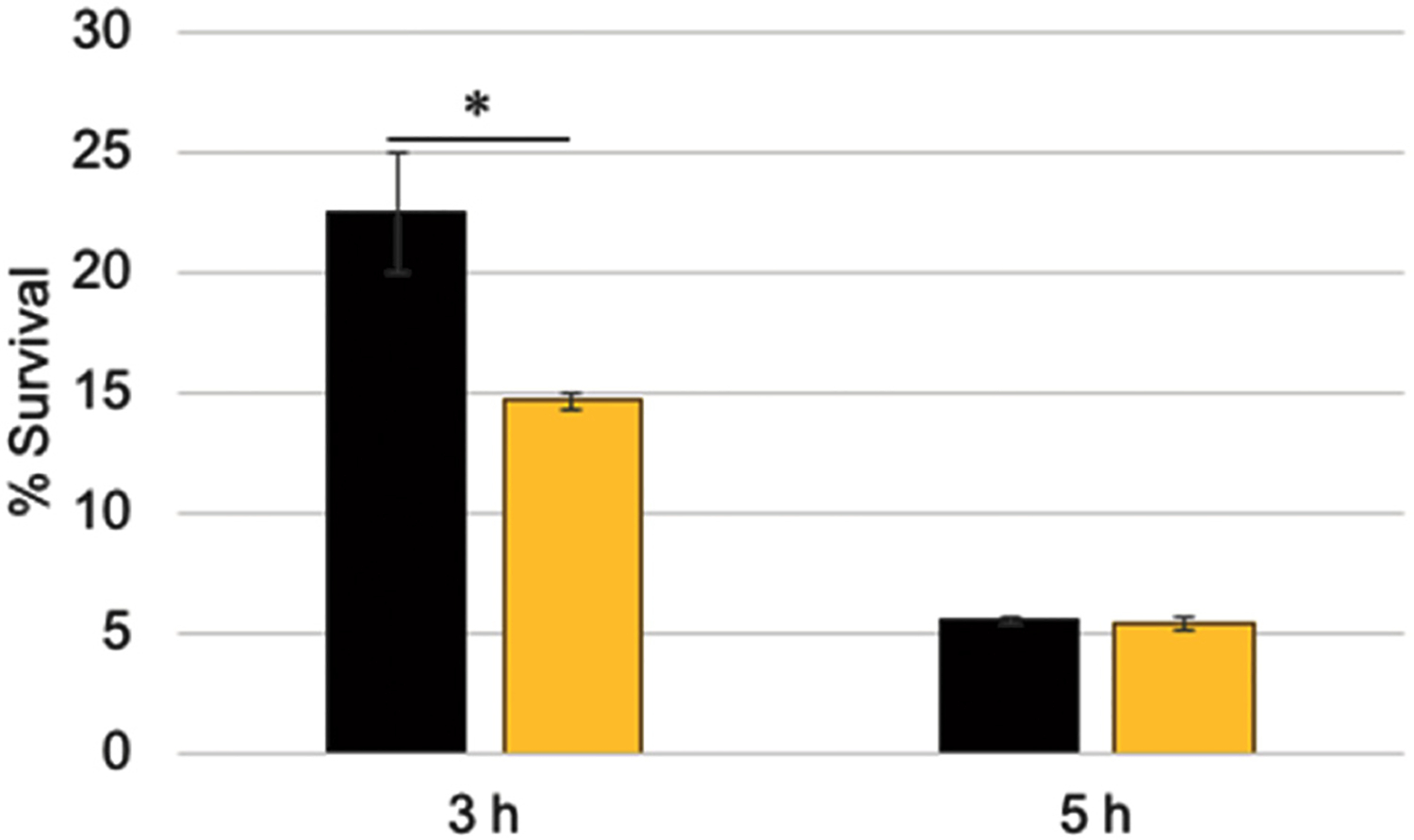
*P. aeruginosa* PAO1 and *ΔpaCAγ1* strains interactions with macrophages. The percent survival in J774A.1 murine macrophages was calculated after a 3 and 5 h infection period utilizing a Gm protection assay. Statistical significance was determined using a single factor ANOVA; * indicates *p* < 0.05.

Previous reports have highlighted that CA activity may support bacterial survival within a host [91]. Based on this and considering the primary function of CAs, *i.e*. the reversable hydrolysis of CO_2_ to bicarbonate, which may help buffer cellular pH under acidic conditions [4], we hypothesized that PaCAγ1 contributes to maintaining intracellular pH and enhances the survival of *P. aeruginosa* within the acidic environment (pH ~5.4) of the phagosome [89]. To test this hypothesis, J774.1 murine macrophages were infected with *ΔpaCAγ1* alongside WT, and the corresponding survival was evaluated at 3 and 5 hpi by using a Gm protection assay. At 3 hpi the deletion of *paCAγ1* resulted in a 25 % reduction of the bacterial survival compared to the WT strain. This difference was no longer observed at 5 hpi, likely due to a low (only 5 %) survival observed at this point for both strains (Fig. 8). These data suggest that PaCAγ1 contributes to the early adaptation and survival of *P. aeruginosa* within macrophages, possibly by alleviating pH related stress.

### 3.9. Biofilm formation

**Fig. 9.**
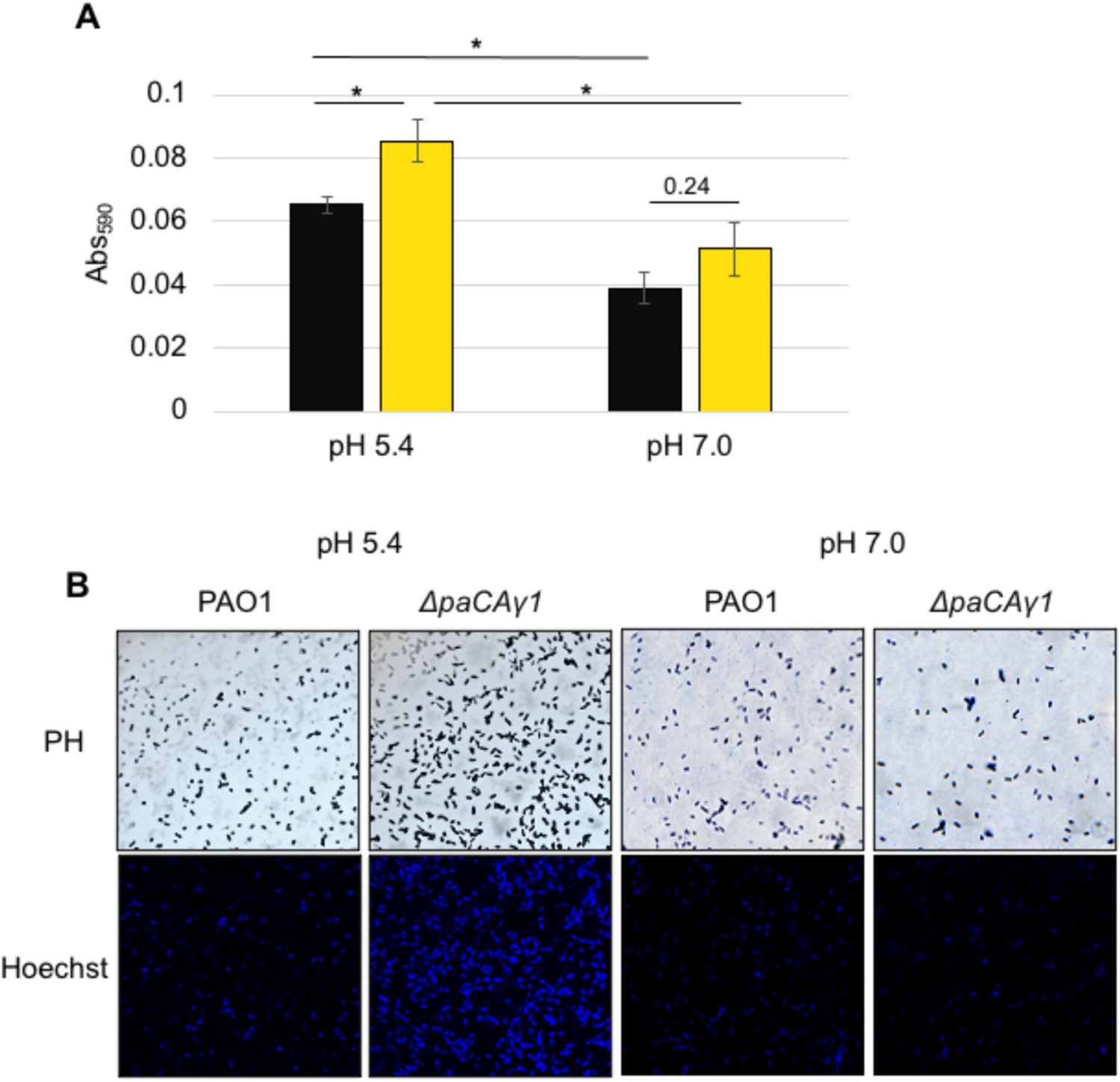
PAO1 and *ΔpaCAγ1* biofilm formation. Cells were grown in BMM8 supplemented with 0.5 mM glycerol statically for 18 h at pH of 5.4 or 7.0. (A) Total biofilm biomass for PAO1 (black) and *ΔpaCAγ1* (yellow) determined by crystal violet staining. Four biological replicates were included in each experiment. Statistical significance was determined using a single factor ANOVA, * indicates *p* < 0.05. (B) Representative images of PAO1 and *ΔpaCAγ1* obtained by fluorescent microscopy: phase contrast (PH) and Hoechst staining. Each experiment included three biological replicates with 10 representative images taken for each replicate.

Considering that biofilm formation is one of the key factors enabling *P. aeruginosa* colonization and survival in a host [92], we investigated the role of PaCAγ1 in the initiation of biofilm formation. For this both WT and *ΔpaCAγ1* were grown in BMM8 supplemented with 0.5 mM glycerol at pH 5.4 and 7.0. According to the CV assay, *ΔpaCAγ1* formed about 35 % more biofilm at pH 5.4 and only slightly more biofilm at pH 7.0 than WT (Fig. 9A). In support, by using fluorescence microscopy, we observed a more substantial surface coverage in the mutant as compared to WT at pH 5.4 (Fig. 9B). These results are consistent with the previous report suggesting such response as an adaptation to the acidic lung environment during cystic fibrosis [93]. However, since biofilm formation is a well-established stress response that enhances bacterial survival [94–96], these results may also indicate that acidic pH introduces a stress that promotes biofilm formation in WT PAO1. The deletion of *paCAγ1* may further increase *P. aeruginosa* susceptibility to acidic stress and, thereby, enhance biofilm formation as a survival mechanism.

## 4. Discussion

There is a significant effort underway to search for novel antibacterial therapies, driven by the critical problem of drug resistance that has emerged in recent decades [97,98]. The ongoing investigations involve attempts to identify novel targets followed by their validation through *in vitro* and *in vivo* experiments. Here, we focused on *P. aeruginosa* due to its critical clinical significance and the increasing frequency of its antibiotic resistance [99]. In particular, we identified and studied a novel CA from this bacterium, designated PaCAγ1, that belongs to the scarcely investigated γ-class. While several studies have focused on the β-CAs of *P. aeruginosa*, to date no γ-CA from this organism has been fully characterized from a biochemical, kinetic, or functional perspective. These data are essential to evaluate the potential of bacterial CAs as alternative targets for antibacterial therapy.

The gene encoding PaCAγ1 was identified through sequence alignments against the *P. aeruginosa* PAO1 genome. Then, the protein was recombinantly expressed and purified to a high purity level with a C-terminal His-tag, which did not interfere with folding and putative oligomerization process. Mass spectrometric analysis of PaCAγ1 demonstrated the lack of the first N-terminal methionine, which is common for recombinant proteins produced in *E. coli* [54,100,101]. In agreement with the predicted folding, far-UV CD spectra recorded in the range of 260–190 nm showed features typical of α/β proteins with high content of β-strands. The wide negative band at 208 nm and the positive signal at 195 nm, that was not as pronounced as expected, suggested the presence of 3_10_ helices [81,102], a feature shared with other few γ-CAs [25,103,104]. Moreover, LS-SEC measurements confirmed a trimeric quaternary structure for this protein (Fig. 3).

Both the WAU assay and stopped-flow experiments confirmed that PaCAγ1 is a functional CA that catalyzes the CO_2_ hydration reaction with kinetic parameters similar to those of the human isoform hCA I (Table 2). The multiple sequence alignment of PaCAγ1 with two previously characterized γ-CAs, Cam and CamH, from *Methanosarcina thermophila* (Fig. 2A) revealed that in PaCAγ1 the acidic loop is not conserved supporting the placement of PaCAγ1 within the CamH subgroup of γ-CAs (Fig. 2A) [3]. Also the essential proton shuttle residue E84 and gate keeper residue Y200, which contributes to Cam extended active-site structure that fine tunes catalysis and influences the conformation of the enzyme, are not conserved in PaCAγ1 (Fig. 2A) [3].

PaCAγ1 demonstrated remarkable stability against temperature-induced denaturation, maintaining its fold up to 60 °C and preserving nearly full catalytic activity at this temperature. Interestingly, at higher temperatures of 80 °C and 100 °C, PaCAγ1 exhibited only slight changes in its secondary structure, although it completely lost its enzymatic activity. This apparent discrepancy suggests that the loss of activity may not be due to global unfolding of the protein but rather due to more subtle structural rearrangements. Notably, γ-CAs are characterized by three active sites formed at the interface between adjacent monomers, where the catalytic metal ion is coordinated by three histidine residues: two coming from one monomer and the third from the neighboring one [25,105]. It is possible that, at these very high temperatures, the enzyme undergoes a relaxation of the trimeric structure, leading to a loss of the catalytic function. These results are consistent with those obtained with recombinant BpsγCA from *Bulkoldera pseudomallei*, where the great structural thermal stability of the protein was evident being only slightly affected by temperatures as high as 92 °C [66].

A series of inhibition assays showed that PaCAγ1 can be targeted to abolish its activity. Among the tested inhibitors, the most striking results were obtained for **AAZ** and **MZA** and the corresponding deacetylated precursors **13** and **14**. In these latter cases, a significant increase of selectivity was gained, particularly in comparison to hCA I. This provides an opportunity for further optimization, as shown for **AAZ**, where scaffold optimization allowed for the development of two lead compounds for treating clinical Vancomycin- Resistant *Enterococci* (VRE) [106].

Upon infecting the host, *P. aeruginosa* encounters diverse hostile conditions, including nutritional challenges and acidic environment, for example, in the lungs of cystis fibrosis patients [107] and during phagocytosis [89]. In response, the pathogen triggers complex rearrangements in gene expression and physiology to enable resistance [108]. Previous studies showed that *P. aeruginosa* encodes for three functional β-class CAs, psCA1, psCA2, and psCA3 [19,51,53]. Functional studies demonstrated that one of them, psCA1, contributes to the pathogen’s adaptation to low CO_2_ environment and plays a major role in calcium deposition and biofilm formation [109]. Inspired by these data, here we aimed to characterize the physiological roles of PaCAγ1. Given the key function of CAs in hydrolyzing CO_2_, their known roles in C1 metabolism and pH homeostasis [4,110,111], and the genomic neighborhood of *paCAγ1*, we investigated the role of PaCAγ1 in *P. aeruginosa* fitness and its ability to withstand low carbon concentration and acidic pH. Comparative planktonic growth studies of the wild type PAO1 and Δ*paCAγ1* at different levels of carbon source and pH supported that the enzyme contributes to *P. aeruginosa* fitness under carbon-limiting conditions and may help maintain cellular pH homeostasis in acidic environments. While the observed effects are relatively modest, it is plausible that the presence of the aforementioned three β-CAs could compensate for the absence of PaCAγ1. Interestingly, Δ*paCAγ1* showed increased surface colonization and biofilm formation, particularly at pH 5.4, which may indicate that the lack of *paCAγ1* increases *P. aeruginosa* susceptibility to acidic stress and, therefore, enhances biofilm formation as a stress survival mechanism [94–96]. Further supporting the importance of PaCAγ1 in alleviating pH-related stress, the deletion of *paCAγ1* reduced the survival of the pathogen during the early stages of J774.1 murine macrophage infection. While the effect appears transient, the early contribution of PaCAγ1 to bacterial survival underscores its importance in the initial adaptation during host-pathogen interactions. Further experiments aiming to elucidate the role of PaCAγ1 in the strain lacking all three β-CAs to avoid functional redundancy are currently underway.

To better understand the potential role of PaCAγ1 in pH homeostasis, the stability and the catalytic activity of the enzyme assessed at different pH values was evaluated and showed that the enzyme is stable in the pH range of 7.0 to 8.5 and has an optimal pH for activity at pH 7.4. At pH below 7.0 and above 8.5, PaCAγ1 exhibited a loss of ordered secondary structure and a consistent decrease in CA activity. This discovery, however, is not contradictory to the role of the enzyme in *P. aeruginosa* survival under acidic environmental pH. As highlighted above, under acidic stress, bacterial cells activate multiple protective mechanisms, including the upregulation of CAs, to prevent cytoplasmic acidification, detrimental to cell viability. Collectively, these mechanisms enable the bacterium to maintain a neutral cytoplasmic pH even when exposed to pH 5.5 [112]. Supporting this notion, PaCAγ1 expression has been shown to increase by 40-fold during growth in cystic fibrosis sputum [113], which is characteristically acidic [107]. This suggests that PaCAγ1 can support *P. aeruginosa* survival under acidic stress conditions through increased expression rather than catalytic activity at low pH. The ongoing studies in our laboratory aim to examine the expression profile of *paCAγ1* in response to environmental pH.

## 5. Conclusions

In this study, we have further expanded the research on CAs in *P. aeruginosa* and, in addition to the three previously characterized β-CAs, we identified a fourth functional CA that belongs to the γ-class, here referred to as PaCAγ1. The presence of multiple CAs in *P. aeruginosa* highlights their physiological importance in the pathogen likely reinforcing the bacterial survival at different CO_2_ and pH levels. Considering that γ-CAs are absent in humans and the lack of similarity between human α-CAs and bacterial γ-CAs, the latter represent a promising target for the development of selective inhibitors to combat *P. aeruginosa* infections. A multi-faceted characterization of this enzyme revealed optimal activity at pH ~7.4, structural stability at neutral pH range, and a remarkable thermal stability. In addition, several compounds were found to inhibit its activity. Despite potential compensation by homologous enzymes, the data indicate that PaCAγ1 contributes to *P. aeruginosa* conditional fitness and survival during phagocytosis. Further studies will explore the expression profile of PaCAγ1 in response to environmental pH, define its role during infections *in vivo*, and determine its 3D structure.

## Supplementary Material

1

## Figures and Tables

**Table 1:** Bacterial strains, plasmids, primers, and inhibitors.

Strains, plasmid, primers, inhibitor	Name, sequence (5′ → 3′), structure	Source
PAO1	WT strain, Alg-	This study
Δp*aCAγ 1*	Δp*aCAγ 1*in PAO1 background	This study
*E. coli* DH5⍺	General purpose cloning strain; Δ(lacZ)M15	NEB
*E. coli* SM10	Donor strain used in biparental mating experiments	NEB
pDONRPEX18Gm	Cm^R^ and Ap^R^/Cb^R^, pEX18Ap with Gateway donor site	[114]
pRB003	pDONRPEX18Gm harboring PA5540 deletion insert	This study
PA5540_Up_F	ggggacaagtttgtacaaaaaagcaggctacGATCATGCCAGCGTGCTC	This study
PA5540_Up_R	TTGCATGTTCAGAACTCGTTCTGCAGCTTGCGGATCATGCTGCAC	This study
PA5540_Dn_F	CTGCAGAACGAGTTCTGAACATGCAA	This study
PA5540_Dn_R	ggggaccactttgtacaagaaagctgggtaCTGACGCCGAAGTGGAAAC	This study
PA5540_seq_F	GGCGATGACTTCCAG	This study
PA5540_seq_R	GCGCTCCAGGGTCGG	This study

**Table 2:** Kinetic parameters for the CO_2_ hydration reaction catalyzed by PaCAγ1 enzyme measured at 20 °C in 20 mM TRIS pH 7.4, 10 mM NaClO_4_. Results are compared to α-class hCA I and II measured at 20 °C in 10 mM HEPES pH 7.5, and to β-CA enzymes from *P. aeruginosa* psCA1–3 measured at 20 °C in 10 mM Tris buffer and 20 mM NaClO_4_ pH 8.3. Inhibition data with the clinically used **AAZ** are also presented.

Isozyme	k_cat_ (s^−1^)	k_cat_/KM (M^−1^ × s^−1^)	K_M_ (mM)	*K*_I_ (nM, AAZ)
PaCAγ1	4.7 × 10^5^ ± 1.45 × 10^4^	4.05 × 10^7^ ± 1.32 × 10^5^	11.6 ± 0.36	47.2 ± 2.17
hCA I^a^	2.0 × 10^5^	5.0 × 10^7^	4.0	250
hCA II^a^	1.4 × 10^6^	1.5 × 10^8^	9.3	12
psCA1^b^	1.8 × 10^5^	7.5 × 10^7^	2.4	37
psCA2	NA	NA	NA	NA
psCA3^c^	1.4 × 10^5^ ± 0.12	1.0 × 10^7^ ± 0.1	14.0 ± 1.8	75.9 ± 3.2

NA, not available.

a From ref. [115].

b From ref. [52].

c From ref. [19] psCA3 is active only at pH 8.3.

**Table 3:** Inhibition of PaCAγ1 from *P. aeruginosa* and human hCA I and hCA II with sulfonamides **1–24** and the clinically used CAI drugs (**AAZ-EPC**), as determined by the Stopped-Flow assay [59] and their Selectivity Index (*SI*).

Inhibitor	*K*_*I*_ (nM)^a^			*SI*	
	hCA I	hCA II	PaCAγ1	hCA I/PaCAγ1	hCA II/PaCAγ1
**1**	28,000	300	189 ± 7.11	148.1	1.6
**2**	25,000	240	155 ± 5.23	161.3	1.5
**3**	79	8	132 ± 8.86	0.6	0.1
**4**	78,500	320	234 ± 11.2	335.5	1.4
**5**	25,000	170	561 ± 32.4	44.6	0.3
**6**	21,000	160	266 ± 14.2	78.9	0.6
**7**	8,300	60	325 ± 13.8	25.5	0.2
**8**	9,800	110	697 ± 44.7	14.1	0.2
**9**	6,500	40	3,490 ± 165	1.9	0.01
**10**	7,300	54	9,670 ± 678	0.8	0.01
**11**	5,800	63	3,670 ± 241	1.6	0.02
**12**	8,400	75	6,090 ± 396	1.4	0.01
**13**	8,600	60	72.6 ± 3.43	118.5	0.8
**14**	9,300	19	97.1 ± 5.32	95.8	0.2
**15**	5,500	80	209 ± 9.19	26.3	0.4
**16**	9,500	94	533 ± 25.8	17.8	0.2
**17**	21,000	125	419 ± 17.7	50.1	0.3
**18**	164	46	793 ± 46.2	0.2	0.1
**19**	109	33	1,320 ± 69.1	0.1	0.03
**20**	6	2	244 ± 16.2	0.02	0.01
**21**	69	11	564 ± 32.9	0.1	0.02
**22**	164	46	386 ± 19.3	0.4	0.1
**23**	109	33	828 ± 51.7	0.1	0.04
**24**	95	30	584 ± 22.2	0.2	0.1
**AAZ**	250	12	47.2 ± 2.17	5.3	0.3
**MZA**	50	14	68.4 ± 2.93	0.7	0.2
**EZA**	25	8	126 ± 6.75	0.2	0.1
**DCP**	1,200	38	5,680 ± 304	0.2	0.01
**DZA**	50,000	9	169 ± 10.9	295.9	0.1
**BRZ**	45,000	3	307 ± 13.1	146.6	0.01
**BZA**	15	9	290 ± 14.4	0.1	0.03
**TPM**	250	10	>100,000	<0.003	<0.0001
**ZNS**	56	35	4,170 ± 286	0.01	0.01
**SLP**	1,200	40	>100,000	<0.01	<0.0004
**IND**	31	15	453 ± 20.1	0.1	0.03
**VLX**	54,000	43	>100,000	<0.5	<0.0004
**CLX**	50,000	21	>100,000	<0.5	<0.0002
**SLT**	374	9	356 ± 23.8	1.1	0.03
**SAC**	18,540	5959	13,800 ± 853	1.3	0.4
**HTC**	328	290	6,600 ± 412	0.05	0.04
**FAM**	922	58	2,690 ± 154	0.3	0.02
**EPC**	8,262	917	5,320 ± 298	1.6	0.2

a Mean from 3 different assays, measured using the Stopped-Flow technique (for hCA I and II errors were in the range of ±5–10 % of the reported values).

## Data Availability

This is an open access article under the CC BY license (http://creativecommons.org/licenses/by/4.0/).

## Appendix A. Supplementary data

Supplementary data to this article can be found online at https://doi.org/10.1016/j.ijbiomac.2025.146755.
