# Supplementary material for: Multi-faceted exploration of the novel active γ-carbonic anhydrase PaCAγ1 in the human pathogen Pseudomonas aeruginosa
Source: Int J Biol Macromol. Author manuscript; Available in PMC 2026 Jun 12. (PMC13261364; doi:10.1016/j.ijbiomac.2025.146755)
Supplement: 1 [file NIHMS2180908-supplement-1.docx]

**Multi-Faceted Exploration of the Novel Active γ-Carbonic Anhydrase PaCAγ1 in the Human Pathogen *Pseudomonas aeruginosa***

**Vincenzo Massimiliano Vivenzio ^a,b#^, Reygan Braga^c#^, Alessandro Bonardi^d^ ,Vincenzo Alterio ^a^, Andrea Scaloni^e^, Claudiu T. Supuran^d^, Marianna Patrauchan^c^, Giuseppina De Simone^a^*, Simona Maria Monti^a^***

*^a^* Istituto di Biostrutture e Bioimmagini, CNR, Via Pietro Castellino 111, 80131 Napoli, Italy.

*^b^* Department of Environmental, Biological and Pharmaceutical Sciences and Technologies, University of Campania "Luigi Vanvitelli", 81100 Caserta, Italy.

*^c^* Department of Microbiology and Molecular Genetics, Oklahoma State University, Stillwater, Oklahoma 74078, United States.

*^d^* Department of NEUROFARBA, Section of Pharmaceutical and Nutraceutical Sciences, University of Florence, Polo Scientifico, Via U. Schiff 6, 50019, Sesto Fiorentino, Firenze, Italy.

*^e^* Proteomics, Metabolomics & Mass Spectrometry Laboratory, ISPAAM, National Research Council, 80055 Portici, Italy.

^*^To whom correspondence should be addressed: (GDS) email: giuseppina.desimone@cnr.it, phone number: +39 081 25 34579; (SMM) [simonamaria.monti@cnr.it](mailto:simonamaria.monti@cnr.it), phone number: +39 081 25 34583.

List of the material:

**Figures S1-S4**

**
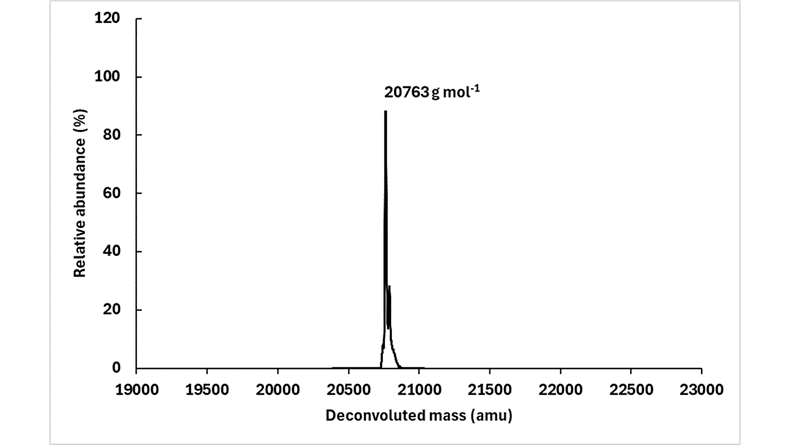
**

**Fig. S1.** Deconvoluted mass spectrum of PaCAγ1


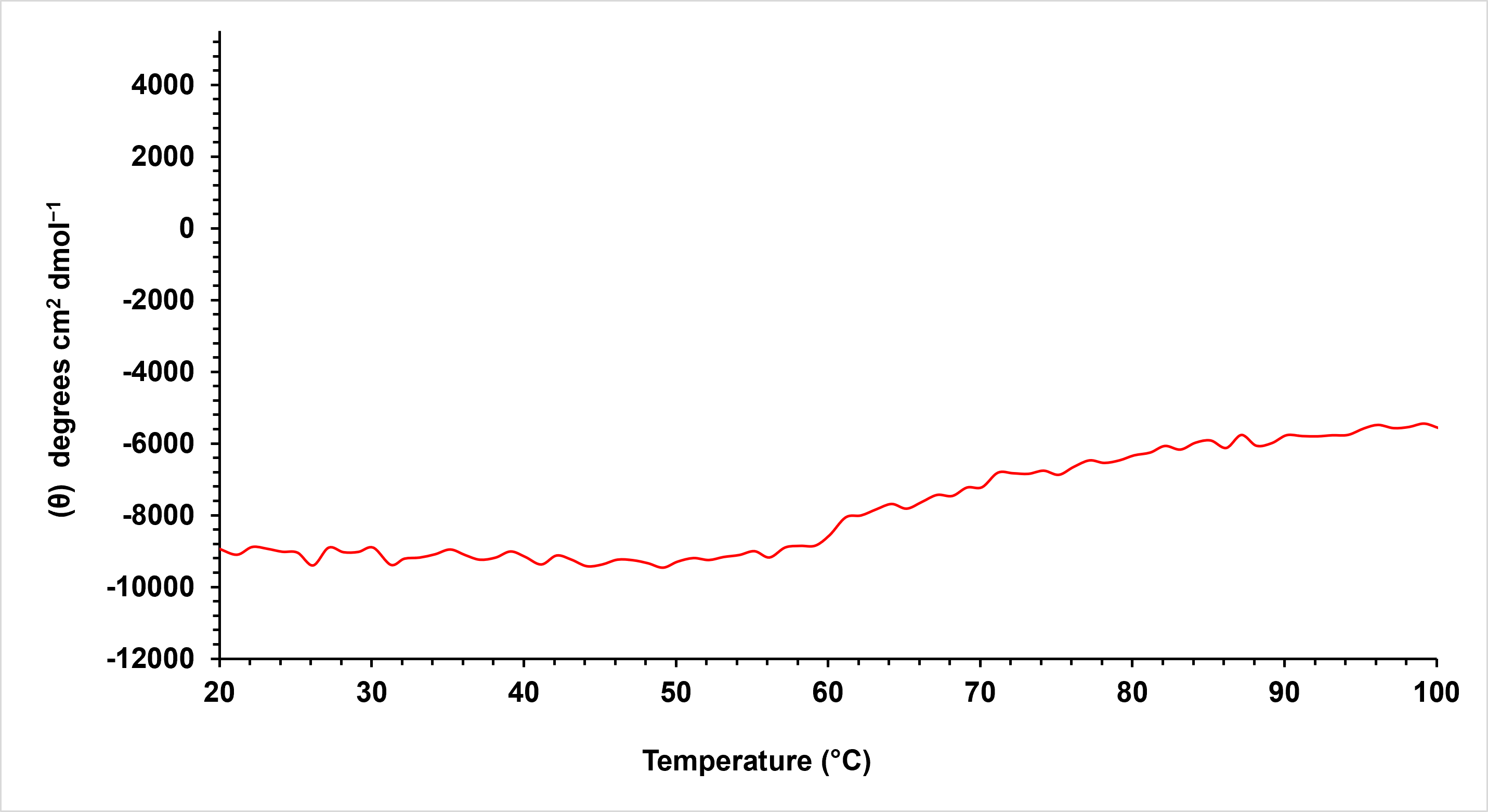


**Fig. S2.** Effect of temperature on PaCAγ1. Variation of ellipticity at 208 nm vs temperature from 20°C to 100°C.

~~

~~

**Fig S3.** **(A),** pH-dependence stability of PaCAγ1 determined by CD; **(B),** pH-dependence of *k_cat_* (s^-1^) for the CO_2_ hydration reaction catalyzed by PaCAγ1 determined by Stopped-Flow spectrophotometric assay.


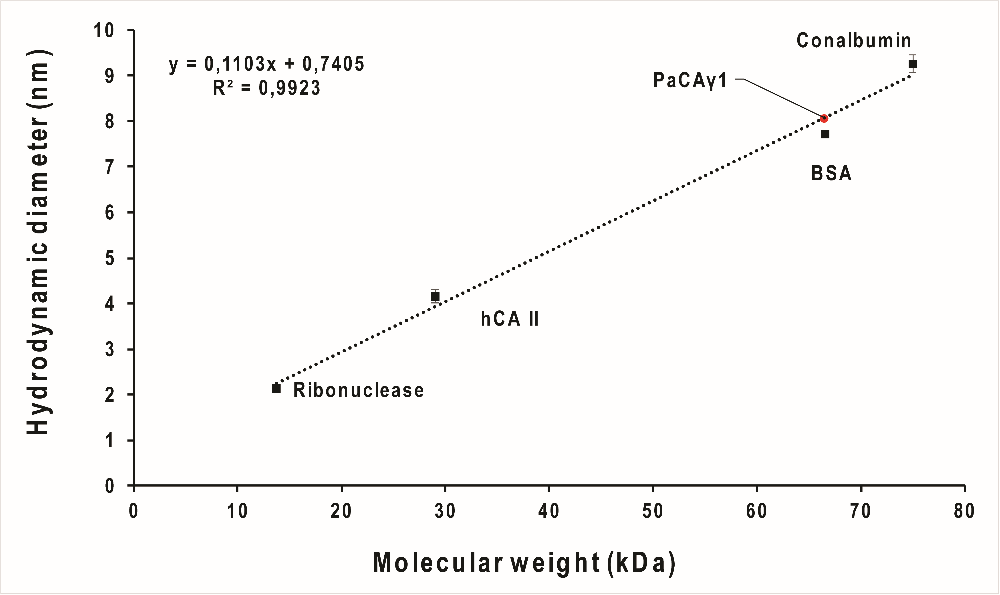


**Fig S4.** Calibration curve of the hydrodynamic diameter (D_H_) as a function of molecular weight (MW) for reference proteins, including bovine serum albumin (BSA), conalbumin, ribonuclease, and human carbonic anhydrase II (hCA II).
